# Tone or tissue? A comparison of trends and risk factors of severe postpartum hemorrhage according to uterine atony or retained tissue in a hospital setting

**DOI:** 10.1371/journal.pone.0318770

**Published:** 2025-02-03

**Authors:** Silje Pettersen, Ragnhild Sørum Falk, Siri Vangen, Lill Trine Nyfløt

**Affiliations:** 1 Norwegian Research Centre for Women’s Health, Oslo University Hospital, Oslo, Norway; 2 Institute of Clinical Medicine, University of Oslo, Oslo, Norway; 3 Oslo Centre for Biostatistics and Epidemiology, Oslo University Hospital, Oslo, Norway; 4 Department of Obstetrics, Drammen Hospital, Drammen, Norway; JSI Research and Training Institute Inc and Addis Continental Institute of Public Health, ETHIOPIA

## Abstract

**Objective:**

To compare trends, outcomes and risk factors for severe postpartum hemorrhage (PPH) due to uterine atony and retained tissue separately.

**Study design:**

This retrospective hospital-based study of severe PPH included deliveries from a 10-year cohort (2008–2017) and a four-year case-control group (2008–2011). Severe PPH was defined as an estimated blood loss of ≥1500 ml or a blood transfusion. Poisson regression was used to estimate the temporal trend in the 10-year cohort. Risk factors were investigated in the case-control group. We performed multinomial regression analysis to investigate associations between pregnancy characteristics and severe PPH caused by uterine atony and by retained tissue compared to controls without severe PPH.

**Results:**

During the 10-year study period, 2.7% of all deliveries were complicated by severe PPH. Uterine atony without concurring retained tissue was the cause in 55.4%, while retained tissue was listed as a cause in 32.2% of the cases. Among women who received ≥ four units of blood products, retained tissue caused 42.6% of cases, and severe PPH resulting in a hysterectomy was caused by retained tissue in 61.2% of cases. The rate of severe PPH caused by uterine atony significantly increased during the study period with an estimated annual percentage change of 8.6%, while the increase in severe PPH due to retained tissue was non-significant. Risk factors associated only with uterine atony were multiple pregnancy, macrosomia, Asian ethnicity and operative delivery, while induction of labor, augmentation of labor, use of anticoagulants and assisted reproduction were associated with both uterine atony and retained tissue.

**Conclusion:**

The observed increased rate of PPH in the study period was mainly driven by an increase in atonic PPH, while the rate of severe PPH caused by retained tissue remained stable. The proportion caused by retained tissue was highest among the most severe cases of PPH. The reason for the increase in severe PPH due to uterine atony, but not retained tissue was not clear, but we speculate that it may be a combination of increasing risk factors with increased awareness of PPH.

## Introduction

Postpartum hemorrhage (PPH) continues to be one of the leading causes of maternal mortality worldwide [1]. While the maternal mortality rate due to PPH is low in high-income countries, [2] the occurrence of PPH is still substantial and there are reports of increasing rates [3, 4]. PPH has usually been defined as an estimated blood loss of 500 ml or more, although this threshold is debated, as a blood loss < 1000 ml may not always be of clinical significance [5]. Severe PPH also lacks a widely accepted definition, with definitions ranging from an estimated blood loss ≥ 1000 ml, to the need for certain medications and procedures, or clinical signs of shock to massive obstetric hemorrhage [6–8].

The main causes or etiologies of postpartum hemorrhage have traditionally been described as fourfold, giving the mnemonic 4 T`s; uterine atony (Tone), genital tract or surgical trauma (Trauma), retained placenta or parts of placental tissue (Tissue) and coagulopathies (Thrombin) [9, 10]. Most studies report uterine atony to be the main cause of PPH, accounting for up to 70%, depending on the definition of PPH and the study population [11–13]. Although PPH may occur in deliveries without risk factors [14], several risk factors have been described. Some risk factors have been associated with specific causes, e.g. previous PPH and multiple pregnancy with uterine atony [15], and previous uterine surgery with placenta accreta spectrum disorders and thus retained tissue [16, 17].

Over the past two decades, studies have reported an increased frequency of PPH in high-income countries [4, 18–21], but the reason behind this trend is not fully understood. The increase has been attributed to more women experiencing uterine atony than previously [14, 19], while retained placental tissue has been reported to be the main cause in the most severe cases of PPH [22]. There are reports of increased rates of placenta accreta spectrum disorders [23], but this condition is still considered too infrequent to cause an increase in the overall rate of PPH [24].

Furthermore, the rise in PPH has been speculated to be associated with certain risk factors, such as induction of labor, advanced maternal age and delivery by cesarean section [25, 26]. Risk factors for PPH have often been described as either independent of cause, or as risk factors for atonic PPH [15]. Risk factors for retained tissue, independent of PPH, have been reported in studies [27, 28], and some of these risk factors are increasing, such as previous cesarean delivery [29]. Many studies on trends and risk factors for PPH are register studies, and we believe that hospital studies can contribute to explore these trends according to cause.

The aim of this study was to evaluate the individual impact of the two main causes of PPH, uterine atony, and retained placental tissue, on the trend of severe PPH. Additionally, we sought to describe the use of blood transfusions, invasive management techniques, and identify risk factors associated with each cause.

## Material and methods

### Study population

**Fig 1:**
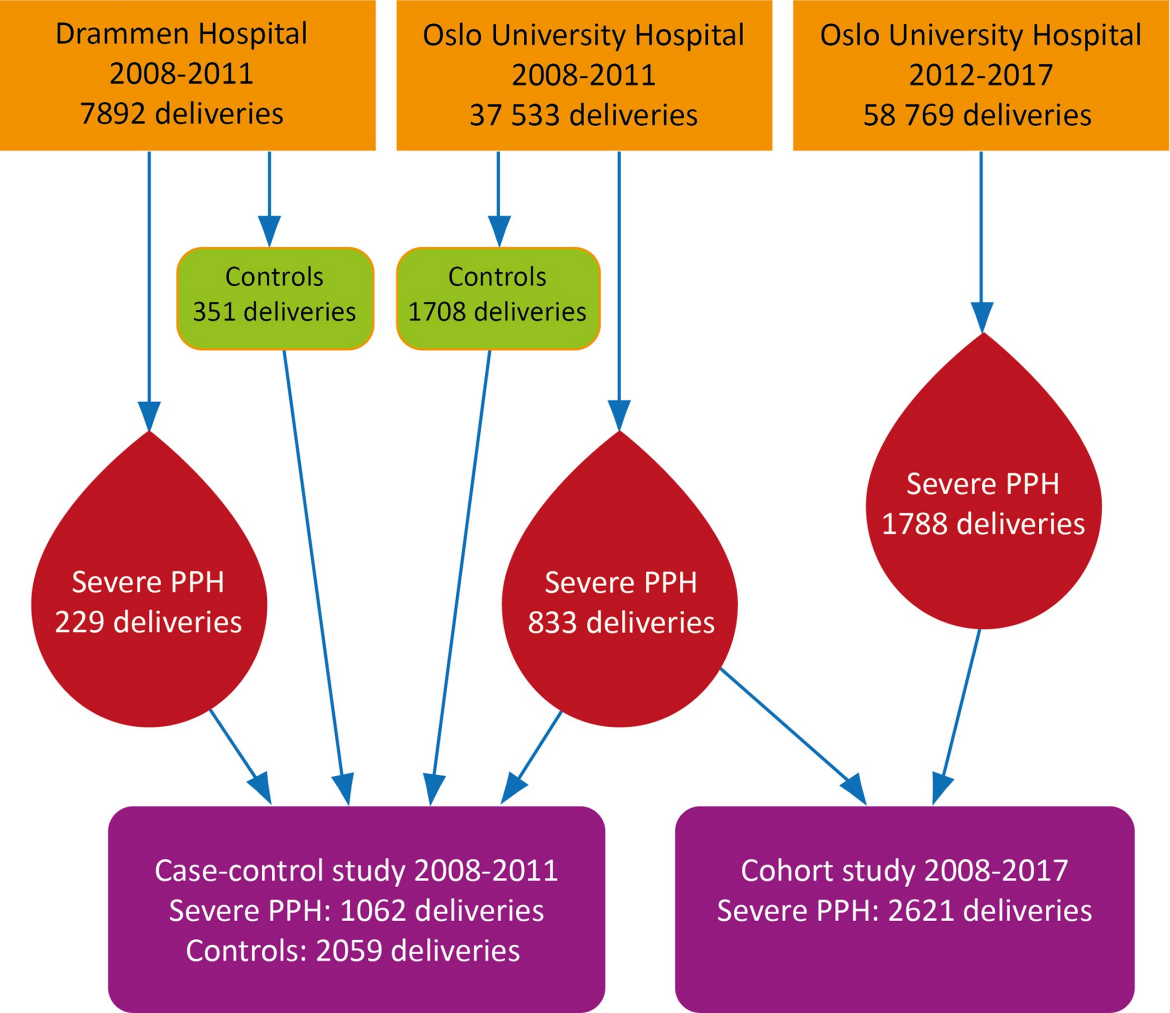
Flow chart.

This retrospective hospital-based study of women with severe PPH from Norway consisted of a cohort study from the years 2008–2017, and a case-control study from the years 2008–2011. Our aim was to combine the results from the case control study with the cohort study of trend according to cause of severe PPH. Women who delivered at Oslo University Hospital in 2008–2017 were the source population for the cohort study, while women who delivered in Oslo University Hospital and Drammen Hospital from 2008 through 2011 were the source population for the case-control study (Fig 1). Accordingly women who delivered between 2008 and 2011 at Oslo University hospital was included in both the cohort and the case control study. Oslo University Hospital is a referral hospital with two locations in the central Oslo while Drammen Hospital is a referral hospital located 30 minutes outside of Oslo. The women giving birth in these hospitals included both high risk and low risk pregnancies, and included the whole pregnant population in the specific area. There are no private hospitals for maternal care in Norway.

### Outcomes and causes

Severe PPH defined as a registered blood loss of 1500 ml or more or transfusion of blood products due to postpartum hemorrhage before discharge from postnatal ward was the main outcome. All cases were identified through hospital databases and patient records. In both hospitals, the attending midwife or physician estimated the blood loss visually, or in combination with metrical measurements. Transfusion of blood products were registered as number of administered units, either collectively or separately as units of red blood cells and units of plasma. Maternal near miss was defined as blood transfusion of ≥ six units of red blood cells, embolization of pelvic arteries, or peripartum hysterectomy. Invasive management of postpartum hemorrhage included curettage, uterine balloon tamponade, uterine compression sutures, embolization of pelvic arteries, and hysterectomy. Causes of severe PPH were classified as uterine atony (Tone), retained placenta or parts of placenta (Tissue), trauma to the birth canal, uterine rupture, or surgical trauma (Trauma) and coagulopathy (Thrombin). In some cases, women were registered with overlapping causes. To examine rates, trends and risk factors according to uterine atony or retained placenta, we selected the cases with uterine atony or retained tissue as causes of severe PPH and divided them into two exclusive groups: 1) severe PPH due to uterine atony without retained placental tissue; 2) severe PPH due to retained placental tissue, with or without coexisting atony.

### Cohort study

To estimate the trend and describe invasive management and outcomes, a 10-year cohort study (2008–2017) at Oslo University Hospital was conducted. A total of 96 313 deliveries constituted the background population. Among these deliveries, 2621 were registered and confirmed as a severe PPH. We registered detailed information on the causes of severe PPH, blood transfusions and invasive management. Further details of the cohort study are previously presented in a separate publication [30].

We estimated the rate of severe PPH per 1000 deliveries for the whole study period, and for each year, caused by uterine atony and retained tissue separately. The exact Poisson method was used to calculate 95% confidence intervals (CI). Poisson regression analysis was performed to estimate trends, where the annual percentage change with 95% CI´s, assuming linear trend, were estimated for each cause separately. We used BioVenn to visualize cases with overlapping causes in a Venn diagram [31]. Furthermore, we calculated the proportions of uterine atony and retained tissue among the blood transfusions and cases with invasive management as percentages and visualized the results graphically as bar charts.

### Case-control study

The case-control study was conducted to assess risk factors for severe PPH [32]. The background population was women who gave birth at Oslo University Hospital or Drammen Hospital from 2008 through 2011, and the total number of deliveries were 43 105. We registered 1062 cases with severe PPH, and included 2059 randomly selected controls (Fig 1). The random controls originated from the same source population, within the same time period and after removing the cases of severe PPH. Manually scrutinizing of the hospitals´ databases and medical records was performed retrospectively to collect detail information of included women. Variables were divided into pre-pregnancy factors, current pregnancy conditions and intrapartum factors. The included characteristics were age at delivery, parity, country of origin, body mass index (BMI), gestational age at delivery, uterine anomaly, uterine surgery, previous cesarean section, previous severe PPH, multiple pregnancy, assisted reproduction, anemia, gestational diabetes mellitus, uterine fibroma, polyhydramnios, placenta previa, use of anticoagulants in pregnancy, severe preeclampsia, mode of delivery, premature rupture of membranes, intrapartum fever, augmentation of labor, induction of labor, and birthweight ≥ 4500 g (macrosmia).

Maternal and obstetric characteristics are presented as frequencies and proportions, for cases (by cause) and controls separately. We performed multinomial regression analysis to explore associations between pregnancy characteristics and severe PPH caused by uterine atony and retained tissue, using the control-group as the reference. The inclusion of variables for the multivariable analysis was based on an outline of a conceptual model together with a directed acyclic graph (DAG). We chose to exclude gestational age, uterine anomaly, uterine surgery, gestational diabetes mellitus and placenta previa from the multivariable analysis because they were closely associated with other variables. No multicollinarity was observed among the included variables and all the variance influence factor (VIF) was less than 1.5. Results are presented as adjusted odds ratios (OR) with 95% CIs and accompanying p-values.

We used Stata (version 17) to perform statistical analyses (StataCorp. 2021. *Stata Statistical Software*: *Release 17*. College Station, TX: StataCorp LLC). Associations with a significance level of ≤0.05 were considered statistically significant.

### Data collection and ethical aspects

This retrospective study was granted a waiver of individual informed consent by the South-East Regional Ethics Committee, reference number 2010/109a. The study was exempted from informed consent to ensure the inclusion of important groups of women, and because the waiver would not affect the rights and welfare of the included women. The data for the case-control study was collected from hospital records by two researchers (Lill Trine Nyfløt and Silje Pettersen) throughout the year of 2012. The data for the remaining years of the cohort study, 2012–2017, were collected by one reaercher; Silje Pettersen throughout the year 2019. During the collection of data the researcher were able to identify the individuals. We de-identified all information obtained from the women’s medical records prior to statistical analysis, and observed strict confidentiality in all stages of the study.

## Results

**Fig 2:**
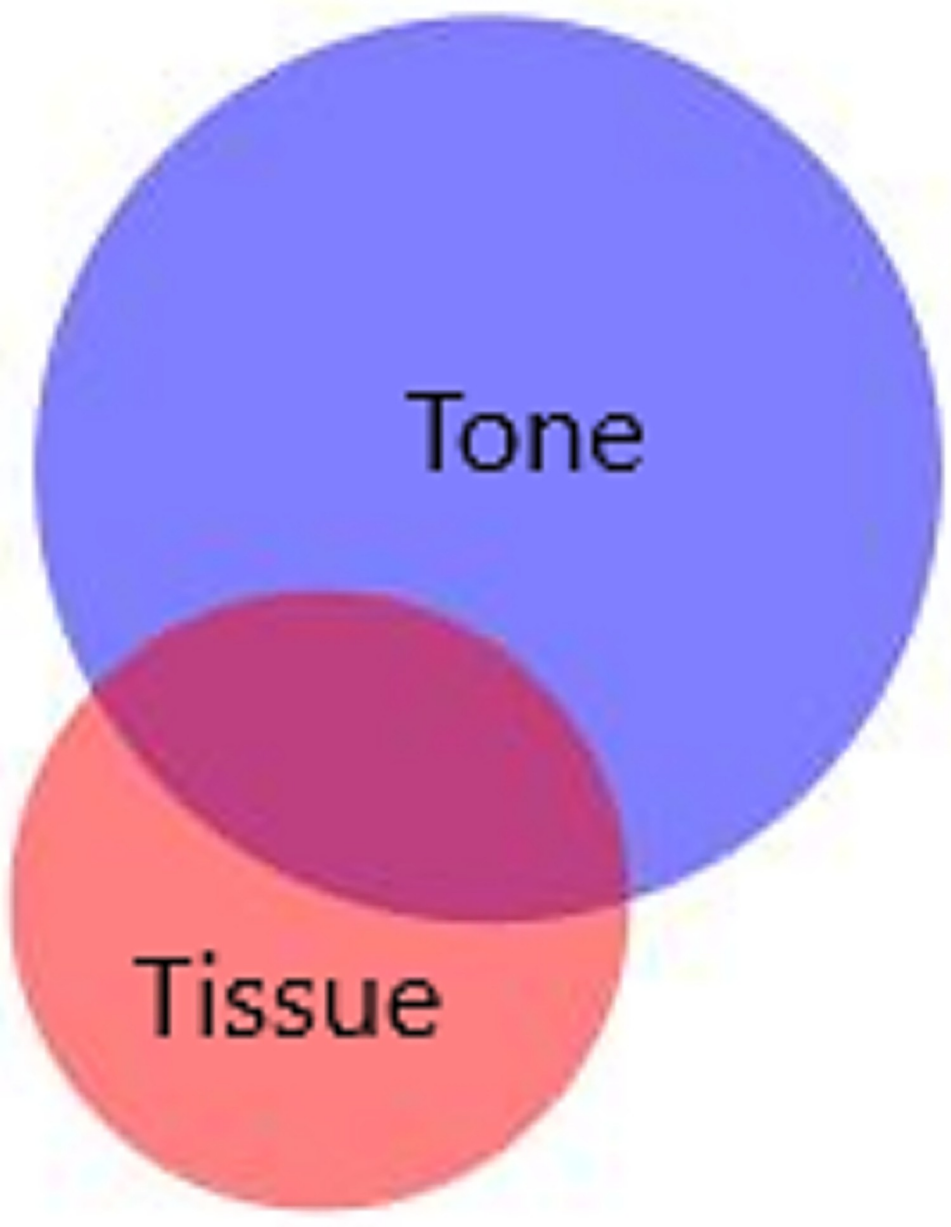
Atony of the uterus (n = 1806) and remaining placental tissue (n = 843) as causes of severe postpartum hemorrhage. Overlapping causes in 345 cases.

Among the 96 313 deliveries in the 10-year cohort study at Oslo University Hospital, 2.7% (2621/96 313) were registered with severe PPH. During the study period, the rate significantly increased from 17.1/1000 in 2008 to 34.2/1000 in 2017, yielding an estimated annual percentage change of 6% (95% CI 4.6–7.5). Among all women with severe PPH, 68.9% (1806/2621) were registered with uterine atony and 32.2% (843/2621) with retained tissue. In 345 cases (13.2%), retained tissue overlapped with uterine atony (Fig 2). More than half of the women (55.4%, 1451/2621), were diagnosed with uterine atony without concurring diagnosed retained tissue. The remaining 328 women (12.5%, 328/2621) did not have either atony or retained tissue among the registered causes of PPH.

**Fig 3:**
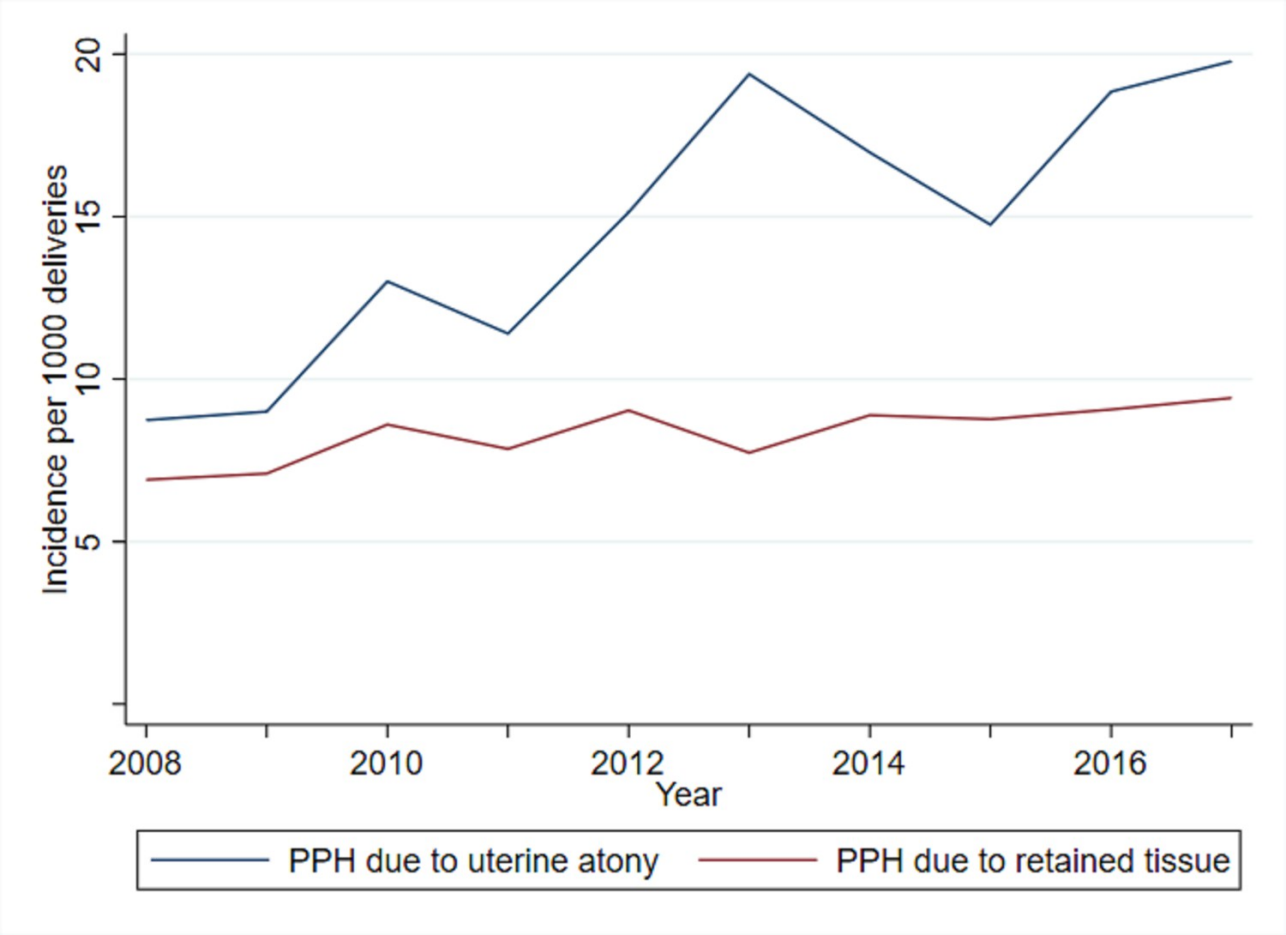
Trend of severe PPH due to the causes uterine atony and retained tissue at Oslo University Hospital (n = 96 313).

The separate trends of severe PPH caused by uterine atony and retained tissue during the 10-year study period are presented in Fig 3. Severe PPH due to atony significantly increased during the study period with an estimated annual percentage change of 8.6% (95% CI 6.6–10.6), while the increase in PPH due to retained tissue was non-significant with an estimated annual percentage change of 2.9% (95% CI 0.4–5.4).

**Fig 4:**
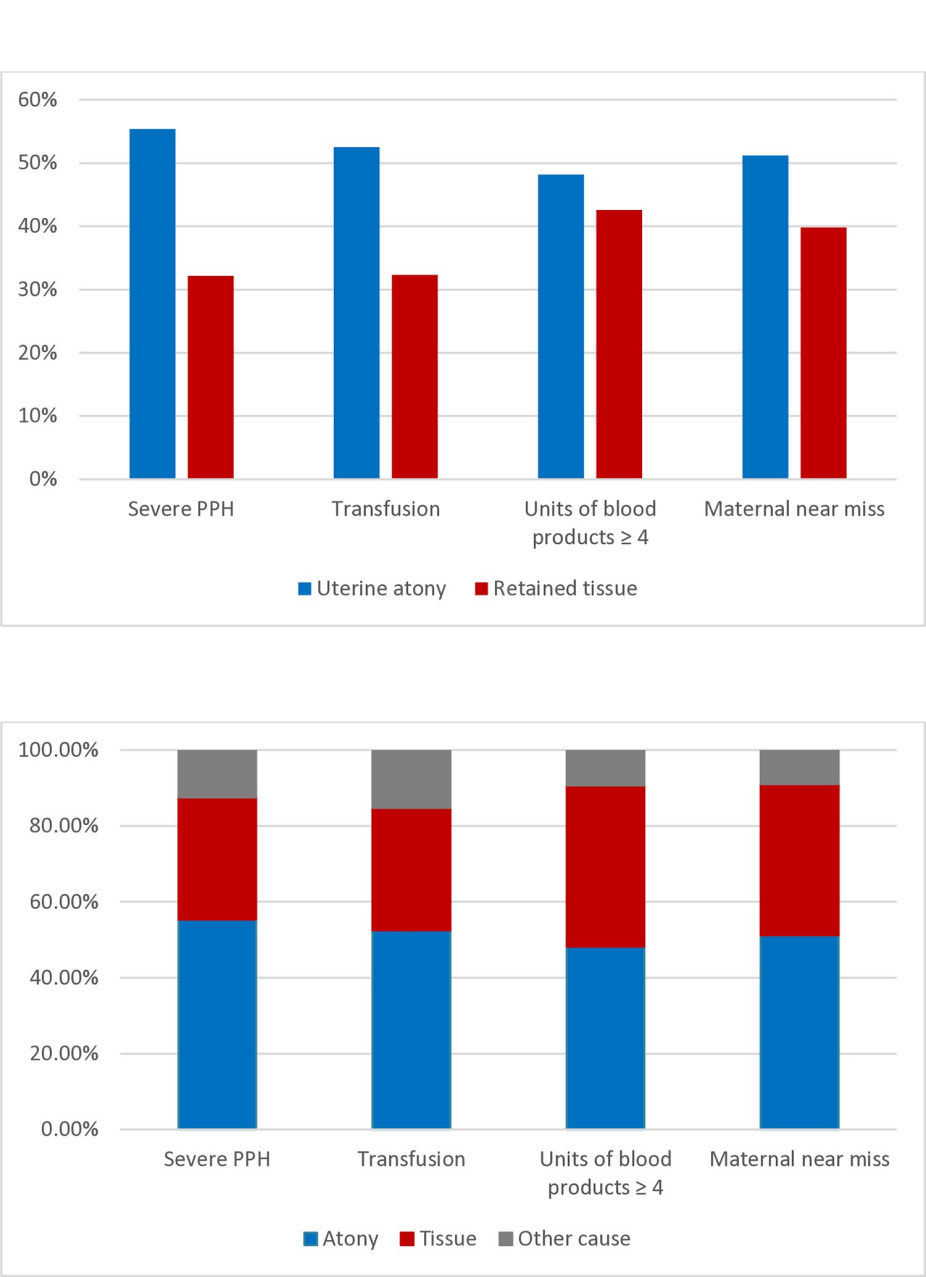
Proportion of severe PPH caused by uterine atony and retained tissue according to blood transfusions and maternal near miss.

Among women who received blood products, but less than four units, the proportion of retained tissue as the cause was 27.2% (368/1355), while in women who received ≥ four units of blood products, the proportion was 42.6% (289/679). The proportional contributions of uterine atony and retained tissue according to the number of blood transfusions, units of blood transfused, and maternal near miss are presented in Fig 4.

**Fig 5:**
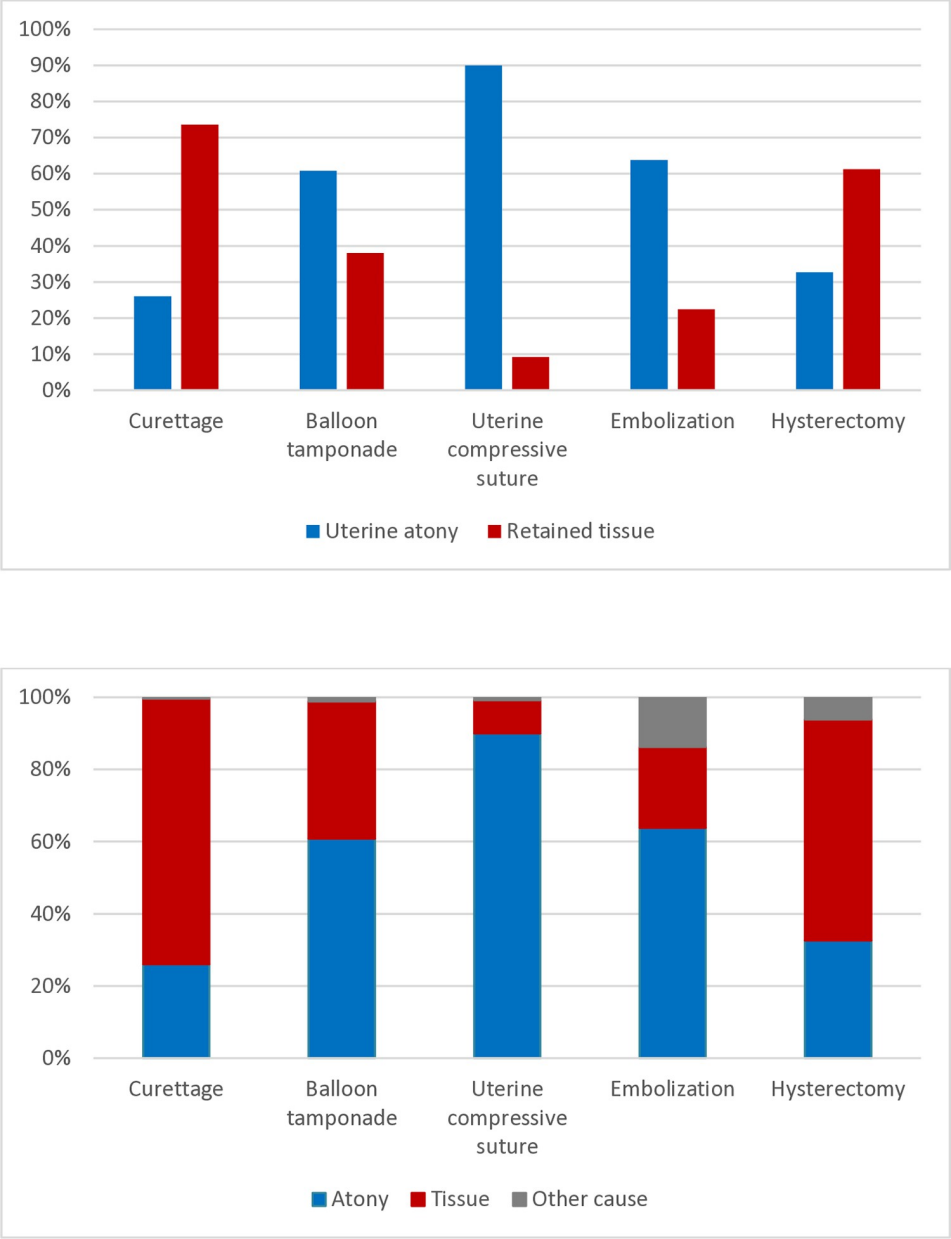
Proportion of severe PPH caused by uterine atony and retained tissue according to invasive management.

The contribution of uterine atony and retained tissue to various invasive managements is presented in Fig 5. Curettage of the uterine cavity was performed in 34.7% of cases, and 73.6% had retained tissue as a cause of hemorrhage. Compressive sutures of the uterus were applied in 4.6% of cases and 90% were caused by uterine atony. Hysterectomies were done in 1.9% of cases, and was most often needed in cases caused by retained tissue (62.1%). S1 Table shows transfusions and management according to the two causes and contains the background data for Figs 4 and 5.

From the case-control study; the total rate of severe PPH during these four years was 2.3%, including a rate of severe PPH due to uterine atony at 1.2% and severe PPH due to retained tissue at 0.8%. The characteristics of women with severe PPH due to uterine atony or retained tissue, and women in the control group are presented in Table 1. The proportion of women aged 40 years or older was higher among women with retained tissue compared to women with uterine atony (8.7% vs. 6.5%). However, in the multivariate analysis the association with age was not statistically significant (Table 2). Uterine atony was more common in primiparous women compared to parous women (60.6% vs. 51.1%), reflected in a significant adjusted OR. (Table 2).Women originating from Asia and Sub-Saharan Africa were overrepresented, especially among cases with severe PPH due to uterine atony (Table 1). However, only women from Asia had a significantly increased risk of severe PPH due to uterine atony in the multivariable analysis (Table 2).Previous severe PPH, anemia, use of anticoagulants, uterine fibroma, assisted reproduction and intrapartum fever were all associated with an increased risk of both uterine atony and retained tissue in the adjusted multivariable analysis (adjusted ORs > 1.6, p <0.05).

**Table 1:** Characteristics of women with severe postpartum hemorrhage (PPH) in the case control study, severe PPH due to atony and severe PPH due to tissue versus controls without severe PPH.

	Controls (n = 2059)	Severe PPH^a^ (n = 1062)	Severe PPH due to uterine atony^b^ (n = 553)	Severe PPH due to retained tissue (n = 378)
	n (%)	n (%)	n (%)	n (%)
**Age at delivery** (years)				
≤19	13 (0.6%)	12 (1.1%)	7 (1.3%)	1 (0.3%)
20–29	652 (31.7%)	299 (28.2%)	166 (30.9%)	90 (23.8%)
30–39	1271 (61.7%)	677 (63.7%)	344 (62.2%)	254 (67.2%)
≥40	123 (6.0%)	74 (7.0%)	36 (6.5%)	33 (8.7%)
**Parity**				
0	1007 (48.9%)	621 (58.5%)	335 (60.6%)	193 (51.1%)
1	738 (35.8%)	295 (27.8%)	144 (26.0%)	129 (34.1%)
2	229 (11.1%)	96 (9.0%)	42 (7.6%)	45 (11.9%)
≥3	85 (4.1%)	50 (4.7%)	32 (5.8%)	11 (2.9%)
**Region of origin**				
Europe/USA/Oceania	1682 (81.7%)	836 (78.7%)	415 (75.0%)	326 (86.2%)
Middle-East/North-Africa	122 (5.9%)	50 (4.7%)	33 (6.0%)	10 (2.6%)
Latin-America	22 (1.1%)	14 (1.3%)	8 (1.4%)	4 (1.1%)
Asia	151 (7.3%)	99 (9.3%)	65 (11.8%)	20 (5.3%)
Sub-Saharan Africa	82 (4.0%)	63 (5.9%)	32 (5.8%)	18 (4.8%)
**Body mass index** (kg/m^2^)				
< 18.5	79 (3.8%)	40 (3.8%)	20 (3.6%)	14 (3.7%)
18.5–24.9	1325 (64.4%)	642 (60.5%)	323 (58.4%)	244 (64.6%)
25.0–29.9	372 (18.1%)	202 (19.0%)	112 (20.3%)	57 (15.1%)
30.0–34.9	144 (7.0%)	83 (7.8%)	49 (8.9%)	28 (7.4%)
≥ 35	50 (2.4%)	31 (2.9%)	19 (3.4%)	8 (2.1%)
**Gestational age** (weeks)	^c^one missing			
< 28	17 (0.8%)	20 (1.9%)	8 (1.4%)	9 (2.4%)
28.0–33.6	36 (1.7%)	40 (3.8%)	22 (4.0%)	9 (2.4%)
34.0–36.6	115 (5.6%)	77 (7.3%)	49 (8.9%)	14 (3.7%)
37.0–40.6	1352 (65.7%)	569 (53.6%)	294 (53.2%)	196 (51.9%)
≥ 41	538 (26.1%)	356 (33.5%)	180 (32.5%)	150 (39.7%)
**Pre-pregnancy**				
Uterine anomaly	13 (0.6%)	16 (1.5%)	4 (0.7%)	10 (2.6%)
Uterine surgery	11 (0.5%)	19 (1.8%)	9 (1.6%)	8 (2.1%)
Previous cesarean section	221 (10.7%)	126 (11.9%)	73 (13.2%)	33 (8.7%)
Previous severe PPH	21 (1.0%)	66 (6.2%)	33 (6.0%)	31 (8.2%)
**Obstetric factors**				
Multiple pregnancy	52 (2.5%)	94 (8.9%)	61 (11.0%)	20 (5.3%)
Assisted reproduction	82 (4.0%)	115 (10.8%)	64 (11.6%)	34 (9.0%)
Anemia	38 (1.8%)	73 (6.9%)	40 (7.2%)	18 (4.7%)
Gestational diabetes mellitus	58 (2.8%)	46 (4.3%)	28 (5.1%)	11 (2.9%)
Uterine fibroma	38 (1.8%)	52 (4.9%)	31 (5.6%)	13 (3.4%)
Polyhydramnios	12 (0.6%)	16 (1.5%)	10 (1.8%)	3 (0.8%)
Placenta previa	3 (0.1%)	56 (5.3%)	37 (6.7%)	16 (4.2%)
Use of anticoagulant	22 (1.1%)	51 (4.8%)	27 (4.9%)	15 (4.0%)
Preeclampsia	28 (1.4%)	48 (4.5%)	25 (4.5%)	7 (1.9%)
**Intrapartum**				
**Mode of delivery**				
Vaginal	1358 (66.0%)	506 (47.6%)	206 (37.3%)	268 (70.9%)
Operative vaginal	251 (12.2%)	211 (19.9%)	103 (18.6%)	77 (20.4%)
Acute cesarean section	245 (11.9%)	248 (23.4%)	180 (32.5%)	17 (4.5%)
Elective cesarean section	205 (10.0%)	97 (9.1%)	64 (11.6%)	16 (4.2%)
Premature rupture of membranes	169 (8.2%)	127 (12.0%)	63 (11.4%)	49 (13.0%)
Intrapartum fever	60 (2.9%)	75 (7.1%)	43 (7.8%)	23 (6.1%)
Augmentation	797 (38.7%)	586 (55.2%)	316 (57.1%)	210 (55.6%)
Induction	402 (19.5%)	348 (32.8%)	188 (34.0%)	126 (33.3%)
Birth weight ≥ 4500 g	58 (2.8%)	52 (4.9%)	38 (6.9%)^d^	12 (3.2%)

Characteristics of women with severe postpartum hemorrhage (PPH) in the case control study, severe PPH due to atony and severe PPH due to tissue versus controls without severe PPH.

^a^32 women did not have tissue or atony as cause of severe PPH

^b^Excluded tissue cause (retained placenta, retained parts of placenta and placenta accreta spectrum disorders)

^c^One missing gestational age in control group

^d^One missing birthweight in case due to atony

**Table 2:** Multinomial logistic regression analysis for severe postpartum hemorrhage (PPH) due to uterine atony (n = 553) and severe PPH due to retained tissue (n = 378), both groups compared to controls without severe PPH (n = 2059).

	Severe PPH due to uterine atony		Severe PPH due to retained tissue	
	Adjusted OR (95% CI)	p-value	Adjusted OR (95% CI)	p-value
**Age at delivery** (years)				
≤19	2.70 (0.88–8.27)	0.08	0.64(0.08–5.29)	0.68
20–29	Ref.		Ref.	
30–39	0.92 (0.71–1.18)	0.50	1.21 (0.90–1.62)	0.20
≥40	0.77 (0.47–1.27)	0.31	1.66 (0.97–2.84)	0.06
**Parity**				
0	**1.38 (1.02–1.87)**	0.04	1.07 (0.78–1.48)	0.66
1	Ref.		Ref.	
2	0.83 (0.54–1.27)	0.39	1.14 (0.75–1.74)	0.54
≥ 3	1.52 (0.86–2.70)	0.15	0.71 (0.32–1.55)	0.39
**Region of origin**				
Europe/USA/Oceania	Ref.		Ref.	
Middle-East/North-Africa	0.96 (0.59–1.59)	0.89	0.45 (0.21–0.97)	0.04
Latin-America	2.07 (0.82–5.21)	0.12	1.15 (0.38–3.50)	0.80
Asia	**1.69 (1.17–2.44)**	<0.01	0.71 (0.42–1.21)	0.21
Sub-Saharan Africa	1.08 (0.62–1.87)	0.79	1.10 (0.57–2.14)	0.44
**Body mass index** (kg/m^2^)				
<18.5	1.17 (0.66–2.08)	0.58	1.16 (0.62–2.17)	0.64
18.5–24.9	Ref.		Ref.	
25.0–29.9	1.07 (0.81-1-42)	0.62	0.79 (0.56–1.10)	0.16
30.0–34.9	1.23 (0.83–1.80)	0.30	0.98 (0.61–1.56)	0.92
≥35	1.15 (0.61–2.18)	0.66	0.77 (0.33-1-79)	0.54
Previous cesarean section	1.27 (0.86–1.88)	0.23	1.04 (0.64–1.67)	0.89
Previous severe PPH	**9.24 (4.92–17.3)**	<0.001	**10.6 (5.7–19.9)**	<0.001
Multiple pregnancy	**2.35 (1.48–3.74)**	<0.001	1.53 (0.80–2.93)	0.20
Assisted reproduction	**1.93 (1.26–2.94)**	<0.01	**2.04 (1.25–3.33)**	<0.01
Anemia	**4.96 (2.92–8.45)**	<0.001	**3.03 (1.59–5.77)**	0.001
Uterine fibroma	**2.53 (1.43–4.48)**	0.001	**2.62 (1.26–5.46)**	0.01
Use of anticoagulant	**4.49 (2.33–8.66)**	<0.001	**3.79 (1.82–7.88)**	<0.001
Preeclampsia	1.91 (0.98–3.73)	0.06	2.59 (0.93–7.16)	0.07
**Mode of delivery**				
Vaginal	Ref.		Ref.	
Operative vaginal	**1.84 (1.33–2.55)**	<0.001	0.97 (0.67–1.39)	0.85
Acute cesarean section	**3.22 (2.40–4.33)**	<0.001	0.24 (0.14–0.43)	<0.001
Elective cesarean section	**2.43 (1.61–3.68)**	<0.001	0.46 (0.25–0.84)	0.01
Premature rupture of membranes	1.12 (0.78–1.60)	0.53	1.47 (0.99–2.19)	<0.001
Intrapartum fever	**1.65 (1.04–2.63)**	0.04	**1.99 (1.14–3.48)**	0.02
Augmentation	**1.73 (1.33–2.25)**	<0.001	**1.49 (1.12–1.99)**	<0.01
Induction	**1.62 (1.25–2.09)**	<0.001	**1.66 (1.25–2.20)**	<0.01
Birth weight ≥ 4500 g	**2.55 (1.54–4.24)**	<0.001	1.08 (0.52–2.22)	0.84

Multinomial logistic regression analysis for severe postpartum hemorrhage (PPH) due to uterine atony (n = 553) and severe PPH due to retained tissue (n = 378) compared to controls without severe PPH (n = 2059).

Bold text indicates statistically significant results (*P* < 0.05).

CI, confidence interval; OR, odds ratio.

Uterine distention (multiple pregnancy and birth weight ≥ 4500 g) was significantly associated with severe PPH due to uterine atony, but not to retained tissue. Cesarean sections and operative vaginal deliveries were associated with an increased risk of uterine atony, but not of retained tissue. Induction of labor and augmentation of labor were significantly associated with both atony and retained tissue.

## Discussion

We observed a significantly increased rate of severe PPH due to uterine atony, but not due to retained tissue. Women receiving ≥ four units of blood products were more often diagnosed with retained placental tissue (42.6%) compared to women receiving < four units of blood products (27.2%). Primiparity, originating from Asia, multiple pregnancy, macrosomic fetus, and operative delivery were risk factors only associated with uterine atony. Previous severe PPH, anemia in pregnancy, assisted reproduction, use of anticoagulants, induction and augmentation of labor were all associated with an increased risk of both uterine atony and retained tissue.

In this study, atony caused most of the increase in severe PPH in the study period. We observed a significant increase in the rate of severe PPH due to uterine atony, but not due to retained tissue. Similar findings with increasing rates of atonic PPH have been described in previous studies [20, 25, 33], all reporting various proportions of uterine atony and retained tissue among women with severe PPH. Compared to studies including blood loss ≥500 ml, studies of massive PPH [34] report lower frequencies of uterine atony [14, 20]. This is also reflected in our study as the proportion of severe PPH caused by retained tissue is larger in women transfused with ≥ four units of red blood cells or with a maternal near miss. We found that the proportion of retained tissue increased to more than 40% in the most severe cases of PPH, similar to a Danish study demonstrating a higher proportion of retained placenta among the most severe cases of PPH [22]. This is consistent with our previous publication [30], where we demonstrated that the trend of massive PPH did not increase in the same study period [30].

Researchers suggest the increase in severe PPH is partly due to an increased awareness among birth attendants, changes in management, and improved identification of PPH [3, 30, 35, 36]. However, retained tissue is often related to more specific procedures, such as curettage and manual removal of the placenta, possibly making the diagnosis more accurate, and thus less likely to be affected by improved registration in the same way as atony.

All studied risk factors were associated with uterine atony. Because the increase was two-fold in the atony group compared to the retained tissue group, risk factors only associated with atony should theoretically be the strongest contributors to the increasing trend. However, these risk factors (primiparity, multiple pregnancy, giving birth to a macrosomic baby, and an operative delivery) did not increase during the study period according to The Medical Birth Register in Norway [37].

Factors associated with both causes, are also possible drivers of the increased rate. A strong risk factor associated with both uterine atony and retained tissue was previous severe PPH. This is in line with other studies which have also shown a recurrence risk with both retained placenta [27, 38] and uterine atony [39, 40]. Assisted reproduction and uterine fibromas have been suggested as risk factors specifically for placenta accreta spectrum disorders and retained placenta in previous studies [16, 41, 42]. However, in our study, these were also associated with uterine atony. These risk factors may directly affect the contractibility of the myometrium, or they may indirectly be associated by confounding factors, such as maternal age, ethnicity and multiple pregnancies. A systematic review of risk factors for atonic PPH [15] stated an unclear association between atonic PPH and uterine fibromas, while assisted reproduction was not assessed. However, according to The Medical Birth Register in Norway [37], the number of babies delivered after assisted reproduction increased from 3.2% in 2008 to 4.7% in 2017.

Induction of labor was also associated with both uterine atony and retained tissue. In Norway, labor induction increased from 15.5% in 2008 to 22.7% in 2017. Augmentation of labor may be a proxy for prolonged labor and related labor dystocia, both known risk factors for uterine atony, and therefore a challenging risk factor to assess [43, 44]. A recent study even suggests that there is no association between use of oxytocin for augmentation of labor and PPH when controlling for confounders [45]. However, it has also been suggested that prolonged labor may affect the expulsion of placenta or parts of placenta, leading to increased risk of retained tissue [27, 38]. Endler et al. also demonstrated an association between prolonged use of oxytocin and retained placenta [27], a possible explanation to why we found that augmentation of labor was associated with both uterine atony and retained tissue. The correlation between augmentation and induction of labor may be stronger with uterine atony than with retained tissue. However, due to our sample size, we were not able to demonstrate this. Thus, a rise in induction and augmentation of labor may have contributed to the increasing trend of severe PPH due to uterine atony in our study.

In summary, uterine atony was the cause responsible for the observed increasing trend of severe PPH when compared to retained tissue. We speculate that both improved awareness and registration, as well as an increase in specific risk factors may have contributed.

Limitations to our study is mainly connected to sample size and generalizability. Especially when analyzing the trend, the number of women included is lower than in registry studies. Also, the analyzed trend is only from the Oslo area and this raises the question if this could be reflected in the whole country. Oslo University Hospital is the largest labor ward in Norway, and the population may differ somewhat from the rest of the country. Additionally, Oslo University Hospital is a referral hospital for the whole country in cases of certain maternal and fetal conditions, and this may limit the generalizability. We may speculate that the non-significant trend of severe PPH due to retained tissue could have been significant with a larger sample size, but still the increase would be less than the increase in severe PPH due to uterine atony. Due to the small number of cases in some of the risk factors in the case-control group, the associated risk factors should be interpreted descriptive, rather than a causal explanation. Another shortcoming is the possible misclassification of severe PPH due to inaccurate blood loss measurement. The estimation of blood loss was done by a combination of visual and metric assessment, and possible over reporting and underreporting is possible. We believe that the thorough registration of variables by two investigators (LTN and SP) limited the misclassifications as women without any record of PPH despite estimated blood loss ≥1500 ml was not included. This limited the inclusion of women with an overestimation of reported blood loss. We also believe that the registration of women with blood transfusion due to PPH avoided underreporting of severe PPH.

The main strength of our study was the confirmation of each case of severe PPH from the hospital database. We collected detailed information on estimated blood loss and the amount of blood products transfused as well as details of invasive management. Additionally for the case-control study there is additional variables collected. Birth registers are usually the database for trend studies, because of the large number of data, but a hospital-based study will contribute as a supplement to these studies. The confirmed cause of the severe PPH is a strength, and gave us the possibility to exclude retained tissue from uterine atony as these sometimes are overlapping causes.

## Conclusion

The trend of severe PPH caused by uterine atony increased significantly during the study period, while we did not observe any increase for severe PPH due to retained tissue. Accordingly, we suggest that the increase in severe PPH was mainly driven by an increase in uterine atony. This could be explained by an increase in certain risk factors associated with atonic PPH, but improved registration and awareness of PPH may also have contributed.

## Supporting information

S1 Table Transfusions and invasive management according to severe PPH caused by uterine atony and retained placenta. (PDF)
