# Supplementary material for: Tone or tissue? A comparison of trends and risk factors of severe postpartum hemorrhage according to uterine atony or retained tissue in a hospital setting
Source: PLoS One. 2025 Feb 3;20(2):e0318770. doi: 10.1371/journal.pone.0318770 (PMC11790139; doi:10.1371/journal.pone.0318770)
Supplement: S1 Table — (PDF) [file pone.0318770.s001.pdf]

Supplemental Table 1. Transfusions and invasive management according to severe PPH caused by uterine atony and retained placenta.

	**Severe PPH** (n=2621)	**Severe PPH due to uterine atony** (n=1451)	**Severe PPH due to retained tissue** (n=843)	**Other cause** (n=328)
	**n (%)**	**n (%)**	**n (%)**	**n (%)**
Transfusion of blood products	2034 (77.6%)	1068 (73.6%)	657 (77.9%)	309 (94.2%)
Transfusion of 1-4 units of blood product (< 4)	1355 (51.7%)	741 (51.1%)	368 (43.7%)	246 (75.0%)
Transfusion of ≥ 4 units of any blood products	679 (25.9%)	327 (22.5%)	289 (34.3%)	63 ((19.2%)
Maternal near miss	211 (8.1%)	108 (7.4%)	84 (10.0%)	19 (5.8%)
Transfusion of ≥ 8 units of red blood cells	85 (3.2%)	44 (3.0%)	34 (4.0%)	7 (2.1%)
Curettage of the uterine cavity	910 (34.7%)	237 (16.3%)	670 (79.5%)	3 (0.9%)
Balloon tamponade of the uterus	268 (10.2%)	163 (11.2%)	102 (12.1%)	3 (0.9%)
Uterine compression sutures	120 (4.6%)	108 (7.5%)	11 (1.3%)	1 (0.3%)
Embolization of pelvic arteries	58 (2.2%)	37 (2.5%)	13 (1.5%)	8 (2.4%)
Hysterectomy	49 (1.9%)	16 (1.1%)	30 (3.6%)	3 (0.9%)
